# Juggling family and professional caring: Role demands, work–family conflict and burnout among registered nurses in Ghana

**DOI:** 10.1002/nop2.178

**Published:** 2018-07-10

**Authors:** Elsie Eunice Amoo Asiedu, Francis Annor, Kwesi Amponsah‐Tawiah, Kwasi Dartey‐Baah

**Affiliations:** ^1^ Department of Organisation & Human Resource Management University of Ghana Business School Legon Accra Ghana; ^2^ Department of Psychology University of Ghana Legon Accra Ghana

**Keywords:** burnout, family demands, work demands, work–family conflict

## Abstract

**Aim:**

Juggling work and family responsibilities remains an important concern for both employees and organizations. This study aimed at examining work and family demands as predictors of work–family conflict and the relationship between work–family conflict and burnout among registered nurses in Ghana.

**Design:**

The study adopted a cross‐sectional survey design.

**Methods:**

Data were collected from a sample of 134 registered nurses selected from five public hospitals in Accra through convenience sampling. Participants responded to structured questionnaires that assessed, among others, experiences of work–family conflict and burnout.

**Results:**

Multiple regression analyses showed that long work hours and weekend schedules were associated with higher levels of work‐to‐family conflict, while the number of older dependants at home was positively related to family‐to‐work conflict. Family‐to‐work conflict was positively related to burnout, while work‐to‐family conflict was not significantly related to burnout.

## 1 INTRODUCTION

Nursing is considered an inherently demanding occupation. Not only are nurses exposed to a variety of job stressors such as pain and death, high emotional expectations of patients, demanding and nonstandard job schedules and work overload but also often lack adequate resources to deal with these stressors (Allen & Mellor, 2002; Demerouti, Bakker, Nachreiner, & Schaufeli, 2000; Lembrechts, Dekocker, Zanoni, & Pulignano, 2015; Van Bogaert et al., 2014). In many developing countries, demands on nurses are exacerbated by shortage in the number of nurses due to factors such as ageing, poor salaries and limited career prospects for nurses (Mason, Leavitt, & Chaffee, 2013; Yildirim & Aycan, 2008). In Ghana, for example, restrictions on public sector employment coupled with the tendency of nurses to migrate to economically developed countries have contributed to a low nurse‐to‐population ratio over the past decade. A recent report published by the Ghana Health Service (GHS) indicates that the nurse‐to‐population ratio in Ghana was 1:959 as at 2014 and has ranged from 1:2,173‐1:959 between 2007‐2015 (GHS, 2015).

Stress emanating from excessive occupational demands has the potential to spill over to nurses' family life, resulting in work–family conflict, “a form of interrole conflict in which the role pressures from the work and family domains are mutually incompatible” (Greenhaus & Beutell, 1985, p. 77). Research has shown that nurses are particularly prone to work–family conflict. In a study in the United States, Grzywacz, Frone, Brewer, and Kovner (2006) found that at least 50% of nurses reported chronic work–family conflict.

Given that the work and family role systems represent the two most important domains of life for most people (Grandey, Cordeiro, & Crouter, 2005), an incompatibility between them can have negative impact on nurses' well‐being and thereby detract from the quality of care rendered and patient outcomes. Extant research has associated work–family conflict with negative physical health outcomes and health‐related behaviours (Allen & Armstrong, 2006; Berkman et al., 2015; van Steenbergen & Ellemers, 2009) as well as psychological outcomes such as anxiety, depression and life satisfaction (Kalliath & Kalliath, 2013; Moen et al., 2015). Work–family conflict also has implications for the quality of work life and retention of nurses. This is evident in previous studies that have linked increased work–family conflict to decreased job satisfaction and organizational commitment and higher turnover intentions (Karatepe & Kilic, 2015; Shockley & Singla, 2011).

Despite its potential impact on the performance and well‐being of nurses, work–family conflict has received less attention in the nursing literature. The present study sought to examine work and family demands as predictors of work–family conflict and to examine the relationship between work–family conflict and burnout among registered nurses in Ghana. The study contributes to the literature in two main ways. First, by focusing on nurses in Ghana, this study extends research on work–family conflict in the nursing context to sub‐Saharan Africa. Extant research on work–family conflict among nurses were conducted mainly in Western and Asian countries. Unlike in Western and Asian countries, research on work–family conflict in sub‐Saharan Africa is in its infancy and little is known about the correlates of work–family conflict among nurses in this geographical context. Cross‐cultural research on work–family conflict underscores the influence of contextual factors in individuals' experiences of work–family conflict (Annor & Burchell, 2017; 2013Ollier‐Malaterre, Valcour, Den Dulk, & Kossek, 2013; Yang, Chen, Choi, & Zou, 2000). Furthermore, nursing practice in sub‐Saharan African countries likely differ from developed countries due to structural differences in healthcare systems. Moreover, the relative absence of formal family‐friendly provisions in both public and private institutions in most countries in sub‐Saharan Africa suggests that balancing work and family responsibilities presents a greater challenge for nurses in sub‐Saharan Africa than in developed countries (Annor, 2016). Thus, nurses' experiences of work–family conflict in sub‐Saharan African contexts may be different from experiences of nurses in other contexts.

Secondly, by examining the link between work–family conflict and burnout, this study also contributes to understanding correlates of burnout in the nursing context. According to Maslach, Schaufeli, and Leiter (2001, p. 399), burnout represents “a psychological syndrome in response to chronic interpersonal stressors on the job.” The demanding nature of nursing makes it “a risk profession for burnout” (Wang, Liu, & Wang, 2015, p. 79). Burnout among nurses has been associated a myriad of negative consequences including anxiety (Khamisa, Oldenburg, Peltzer, & Ilic, 2015), lack of motivation (Wang et al., 2015), reduced quality of care and patient safety (Halbesleben, Wakefield, Wakefield, & Cooper, 2008; Nantsupawat, Nantsupawat, Kunaviktikul, Turale, & Poghosyan, 2016), absenteeism (Gil‐Monte, 2008) and intention to quit (Labrague et al., 2017). Although a plethora of studies have investigated various occupational antecedents of burnout in the nursing literature, few studies have explored the relationship between work–family conflict and burnout among nurses. To the best of our knowledge, no study has examined the link between work–family conflict and job burnout among nurses in Ghana.

## 2 LITERATURE REVIEW AND HYPOTHESES

The concept of work–family conflict is rooted in role stress theory (Goode, 1960), which assumes that individuals have limited resources (time and energy) to expend on multiple role obligations. Consequently, participation in work and family roles deplete these limited resources, leading to role conflict (Kahn, Wolfe, Quinn, Snoek, & Rosenthal, 1964). As defined earlier, work–family conflict occurs when participation in the work (family) role makes it more difficult to participate in the family (work) role (Greenhaus & Beutell, 1985). According to Greenhaus and Beutell (1985), work–family conflict may be time‐based, strain‐based or behaviour‐based. Time‐based conflict occurs when time demands in one role make it difficult to meet demands in another role. Strain‐based conflict occurs when strain resulting from demands in one domain interferes with meeting demands in another domain. Behaviour‐based conflict occurs when specific behaviour patterns that are considered appropriate or desirable in one domain are considered inappropriate or counterproductive in another domain.

In line with the conceptualization of work–family conflict as a bidirectional construct (see Byron, 2005; Michel, Kotrba, Mitchelson, Clark, & Baltes, 2011), the present study distinguishes between work‐to‐family conflict (WFC) and family‐to‐work conflict (FWC). WFC occurs when demands associated with the work role interfere with involvement in the family role, while FWC occurs when responsibilities associated with the family role interfere with meeting demands in the work domain. The distinction between WFC and FWC is important, as both directions of conflict have been found to be associated with different antecedents and consequences (Michel et al., 2011; Shockley & Singla, 2011).

Past research has examined several variables as potential antecedents of work–family conflict. This stream of research suggests that antecedents of WFC and FWC are domain‐specific, such that work domain factors tend to have stronger influence on WFC, whereas family domain factors have stronger influence of FWC (Byron, 2005; Michel et al., 2011). Following this line of research, the present study examined work hours, weekend work and workload as antecedents of WFC, whereas number of children, number of older dependents and time commitment to the family were examined as antecedents of FWC.

In the work domain, work hours have been one of the most frequently studied antecedents of work–family conflict. From the perspective of role theory, time is a limited resource; hence, greater time commitment to the work role detracts from the amount of time available for the family domain. In line with this assertion, several studies have found positive relationship between weekly work hours and WFC (e.g., Adkins & Premeaux, 2012; Dugan, Matthews, & Barnes‐Farrell, 2012). Related to work hours, nonstandard work schedules such as weekend work can impinge on employees' ability to meet family‐related demands. Studies have shown that weekend work can result in higher levels of fatigue and stress, which can undermine individuals' ability to meet demands in the family domain (Fenwick & Tausig, 2001). For example, Fuß, Nubling, Hasselhorn, Schwappach, and Rieger (2008) found that strain resulting from working on weekend was positively related to WFC among physicians. In a study among dual‐earners in three countries (Finland, the Netherlands and the United Kingdom), working nonstandard schedules was found to be associated with increased time‐based WFC (Tammelin, Malinen, Rönkä, & Verhoef, 2017). Given the tendency for nurses to work extremely long hours and on weekends, particularly in developing countries like Ghana, we believe that work hours could constitute an important precursor to WFC in this occupational setting.

As indicated earlier, nurses often respond to high levels of pressure from patients and management. In a context where nurse‐to‐population ratio remains low, as in the case of Ghana, nurses may be faced with high levels of workload in terms of the number of patients they attend to at any given time. In the work–family literature, workload is noted to be a major source of work‐related stress and has been found to be a robust predictor of work–family conflict (Michel et al., 2011). Heavy workloads may increase the amount of time devoted to the work role (Adkins & Premeaux, 2012), thus leaving less time for family‐related tasks. Additionally, excessive workload may result in strain and negative psychological states such as frustration and irritability, which may spill over to the family domain. Accordingly, Yildirim and Aycan (2008) reported a positive relationship between workload and WFC among academic and clinical nurses in Turkey. Similarly, quantitative job demand at work was a positive predictor of WFC in a study among hospital physicians in Germany (Fuß et al., 2008).

In the family domain, time commitment to family responsibilities is viewed as an objective indicator of demands in the family (Parasuraman & Simmers, 2001) and has been associated with higher levels of FWC (Byron, 2005; Michel et al., 2011). In particular, the presence of children at home likely increases time demands in the family domain, thereby increasing the potential for FWC (Parasuraman & Simmers, 2001). However, previous studies have yielded mixed results on the link between the number of children at home and FWC. While some studies found that having a high number of children at home was associated with high levels of FWC (Grzywacz, 2000; Kinnunen & Mauno, 1998), some studies found no relationship between the number of children at home and FWC (Annor, 2016). These mixed findings could be because the number of children at home represents a narrow definition of responsibilities in the family role (Rothausen, 1999). In collectivistic societies such as Ghana, family responsibilities extend beyond one's own children to include responsibility for extended family relatives, especially older parents (Annor, 2014). Empirical evidence suggests that caring for older parents could have stronger negative influence on work performance than caring for children (Kossek, Colquitt, & Noe, 2001). Therefore, in the present study, family demands are conceptualized in terms of time commitment to the family and the number of children and older dependants at home.

As stated earlier, work–family conflict has been associated with several negative personal and organizational outcomes. Among these outcomes, the present study focuses on burnout. Maslach et al. (2001) conceptualized burnout as a multidimensional construct consisting of emotional exhaustion, depersonalization and reduced personal accomplishment. Emotional exhaustion represents feelings of being overextended and depleted by the emotional and physical demands of one's work; depersonalization (cynicism) is characterized by a detached, mentally distanced and cynical response to various aspects of the job; and reduced personal accomplishment (inefficacy) refers to feelings of inadequacy, lack of achievement and productivity in work.

Although earlier research on burnout considered the phenomenon to be mainly work‐related, it is acknowledged that burnout could emanate from interactions between employees' work and nonwork lives (Maslach et al., 2001). There is evidence that conflict between work and family roles can have significant influence on employees' experience of burnout. In a meta‐analytic study on outcomes of work–family conflict, Allen, Herst, Bruck, and Sutton (2000) found that the relationship between work–family conflict and burnout was one of the strongest and most consistent findings. However, Allen et al.'s meta‐analysis focused mainly on one direction of work–family conflict (i.e., WFC). In a recent study among police officers and civilian staff, Haines and Harvey (2013) found that WFC and FWC were positively related to burnout. Moreover, recent meta‐analyses (Amstad, Meier, Fasel, Elfering, & Semmer, 2011; Reichl, Leiter, & Spinath, 2014) suggest that both directions of work–family conflict are related to higher levels of burnout, though the relationship appears stronger for WFC than for FWC.

Based on the literature reviewed and the objectives of the study, the following hypotheses were tested in the present study:

Hypothesis 1: Work demands (i.e., work hours, weekend work and workload) will be positively related to WFC.
Hypothesis 2: Family demands (i.e., time commitment to the family, number of children and number of older dependants) will be positively related to FWC.
Hypothesis 3: Work–family conflict (i.e., WFC and FWC) will be positively related to burnout.

## 3 METHODS

### 3.1 Design and sample

The study was conducted using a cross‐sectional survey design. The participants consisted of registered nurses working full‐time in five public hospitals in Accra, Ghana's capital city. All the hospitals operate under the GHS. The study focused on both male and female nurses who were either married or single, with or without children. It is possible that individuals who are not married may have responsibilities for older parents, making the experience of work–family conflict plausible among them (Annor, 2014).

In all, 134 nurses participated in the study. The sample consisted largely of females (85.8%), a reflection of the nursing profession in Ghana, which is dominated by females. In terms of the level of education, most of the participants (79.8%) had a certificate or diploma in nursing from a nurses' training college and a few (20.1%) had bachelor's degrees or postgraduate qualifications. About two‐thirds (64%) of the participants were married; 77% had at least one child at home; and 56% lived with at least one older dependent. Job tenure was high among the participants, with mean job tenure of 11.6 years (*SD* 10 years). As shown in Table 1, most (52.6%) participants had been in their current jobs for less than 10 years, 19.5%, 14.1% and 10.2% had worked 10–19 years, 20–29 years and 30 years and above respectively. The mean work hours for the nurses was about 44 hr per week and the majority (53%) reported working beyond the statutory maximum of 40 hr per week. Information on participants' demographic characteristics is presented in Table 1.

**Table 1:** Participants' demographic information

Variable/categories	Frequency	Percentage
Gender		
Male	19	14.2
Female	115	85.8
Age		
20–29	49	36.6
30–39	28	20.9
40 and above	57	42.5
Marital status		
Single	47	35.8
Married	86	64.2
Education level		
Certificate/diploma nursing	107	79.9
Bachelor's degree or better	27	20.1
Weekly work hours		
Up to 40 hr	63	47.0
41 hr or more	71	53.0
Job tenure		
1–9 years	72	56.2
10–19 years	25	19.5
20–29 years	18	14.1
30 years or more	13	10.2

### 3.2 Data collection procedure

Permission was sought from the five health facilities for the conduct of the study by means of an introductory letter explaining the purpose of the study. Thirty questionnaire packages were distributed to a contact person in each hospital, who distributed the questionnaires to nurses willing to participate in the study. A covering letter was included in each questionnaire package to explain the purpose of the study to participants and assure them of confidentiality and anonymity. Participants were made aware that participation in the study was entirely voluntary and that they could choose to withdraw at any point. Completed questionnaires were returned to the contact persons within 3 weeks after distribution. Out of the 150 questionnaires, 134 completed questionnaires were returned, representing a response rate of 89%.

### 3.3 Measures

*Work–family conflict* was measured with a 10‐item scale developed by Netemeyer, Boles, and McMurrian (1996). The scale consisted of two subscales, with five items measuring WFC and another five items measuring FWC. A sample item on the WFC dimension of the instrument is “after work I come home too tired to do the things that I would like to do”; and a sample item on the FWC dimension is “I am often forced to make changes in my schedule due to demands at home.” Responses on the items were rated on a five‐point scale ranging from 1 (“strongly disagree”)‐5 (“strong agree”). The measures of WFC and FWC represent the average of the five items on the respective subscales with scores ranging from 1‐5 such that high scores indicate high levels of conflict. The reliability coefficients for WFC and FWC in this study are 0.83 and 0.78 respectively.

*Work demands*. Work hours and weekend work were used together with workload as indicators of work demands. Work hours were measured by asking respondents to indicate the number of hours they typically worked per week in their main job. Participants were also asked to indicate how often they were required to work during weekends on a scale ranging from 1 (“never”) to 4 (“every weekend”). Workload was measured with four items adapted from Aryee, Luk, Leung, and Lo (1999). The participants were asked to rate how often they experienced four conditions reflecting quantitative workload at work. A sample item on this scale is “how often do you have to do more work than you can do?” Responses on the instrument were rated on a 4‐point scale ranging from 0 (“never”)‐3 (“often”). The reliability for the four‐item measure in this study is 0.61.

*Family demands*. Number of children and older dependents at home together with family time commitment were used as indicators of family demands. Participants were asked to indicate the number of children and older relatives living with them at home. Family time commitment was also measured by asking respondents to indicate the number of hours they spent on family‐related tasks in a week.

*Burnout* was measured with 17 items adapted from the Maslach Burnout Inventory—General Survey (MBI‐GS) (Bakker, Demerouti, & Schaufeli, 2002). The MBI‐GS consists of three subscales, which measure the three dimensions of burnout: emotional exhaustion, cynicism and inefficacy. Exhaustion was measured with five items (e.g., “I feel emotionally drained from my work”), cynicism was measured with seven items (e.g., “I have become more cynical about whether my work contributes anything”) and inefficacy was measured with five items (e.g., “I worry that this job is hardening me emotionally”). Participants' responses were rated on a 7‐point scale ranging from 0 (“never”)‐6 (“every day”). A composite burnout score was computed by averaging responses on the three subscales with high scores reflecting high levels of burnout. The alpha coefficient for the composite burnout scale in this study is 0.77.

*Control variables*. Age, gender and marital status were included as control variables in the study, as these have been associated with work–family conflict in previous studies. Age was measured as a continuous variable. Gender and marital status were coded as dichotomous variables (Gender: 1 = male, 2 = female; Marital Status: 1 = single, 2 = married).

### 3.4 Data analysis

The data were analysed using IBM SPSS version 21.0. The analysis was done in three stages. In the first stage, descriptive statistics were conducted to examine the means and standard deviations of the variables in the study, followed by Pearson's product‐moment correlations to examine bivariate correlations among variables in the study. In the second stage, a series of hierarchical multiple regression analyses were conducted to examine the hypothesized relationships. Specifically, each direction of work–family conflict was regressed on the control variables (age and gender) followed by work and family demands. In the third stage, another hierarchical multiple regression analysis was conducted by regressing burnout on both WFC and FWC, while controlling for other variables that were significantly correlated with burnout.

### 3.5 Ethical approval

Approval of the research protocol and ethical clearance for the study were obtained from the University of Ghana Business School. Institutional approval was also obtained from the heads of the selected health facilities. A written informed consent was obtained from each participant and no identifying information was obtained from participants. To enhance anonymity, participants were asked to seal the completed questionnaires in envelopes that were included in the package before handing them to the contact persons in the various health facilities. Obtaining patient consent was not necessary, as the study was based on nurses only.

## 4 RESULTS

Descriptive statistics and zero‐order correlations for the study variables are presented in Table 2. As expected, work hours, weekend work and irregular work schedules were significantly correlated with WFC, while number of older dependants was correlated with FWC. Both WFC and FWC were significantly correlated with burnout.

**Table 2:** Means, standard deviations and zero‐order correlations among study variables

	Variable	Mean	*SD*	1	2	3	4	5	6	7	8	9	10	11	12
1	Gender	—	—	1.00											
2	Marital status	—	—	0.09	1.00										
3	Age	37.59	11.32	0.14	0.61**	1.00									
4	Number of older parents	1.04	1.15	−0.34**	−0.06	−0.06	1.00								
5	Weekend work	2.99	1.07	0.02	−0.04	−0.34**	−0.04	1.00							
6	Number of children	2.27	1.78	0.05	0.61**	0.59**	0.19*	−0.12	1.00						
7	Family time commitment	7.16	5.53	−0.01	0.08	−0.01	0.04	0.11	0.07	1.00					
8	Weekly work hours	44.41	7.69	−0.14	−0.06	−0.04	0.17	0.12	−0.02	−0.19*	1.00				
9	Workload	2.38	0.46	−0.01	−0.11	−0.27**	−0.25**	0.29**	−0.20*	0.09	0.11	1.00			
10	Work‐to‐family conflict	3.28	0.90	−0.02	0.13	−0.13	0.14	0.31**	0.04	−0.09	0.18*	0.13	1.00		
11	Family‐to‐work conflict	2.28	0.79	−0.17*	−0.02	−0.19*	0.40**	0.03	−0.05	0.10	0.02	−0.11	0.38**	1.00	
12	Burnout	1.73	0.83	−0.32**	0.06	−0.19*	0.30**	0.19*	−0.04	0.01	0.08	0.08	0.33**	0.52**	1.00

Note

*p <0.05; **p <0.01.

Table 3 presents results of regression analysis on relationship between work demands and WFC. As shown in Table 3, long work hours (β = 0.17, *p* < 0.05) and working on weekends (β = 0.23, *p* < 0.05) were associated with higher levels of WFC. Contrary to expectation, workload (β = 0.02, *p* > 0.05) was not a significant predictor of WFC. Together, work demands accounted for 8% of the explained variance in WFC beyond age, gender, and marital status. These results provided partial support for Hypothesis 1, which suggested that work demands would be positively related to WFC.

**Table 3:** Multiple regression of the relationship between work demands and WFC

Step	Variable	*B*		*SE*	β	Δ*R* ^2^	*F* for Δ*R* ^2^
1	Controls						
	Gender		0.02	0.22	0.01		
	Age		−0.02	0.01	−0.19*		
	Marital status		0.49	0.19	0.26*	0.08	*F* (3, 130 = 3.75*
2	Work demands						
	Weekly work hours	0.02		0.01	0.17*		
	Weekend work	0.20		0.08	0.23*		
	Workload	0.03		0.17	0.02	0.08	*F* (3, 127) = 4.19**
	Total *R* ^2^	0.13					
	*F* (6, 127)	4.11**					

Note

*p < 0.05; **p < 0.01.

As shown in Table 4, family demands accounted for 15% of the variance in FWC beyond the control variables. From Table 4, number of older dependants (β = 0.43, *p* < 0.01) was a significant positive predictor of FWC. However, number of children (β = −0.13, *p* > 0.05) and time spent on family‐related tasks (β = 0.08, *p* > 0.05) did not significantly predict FWC. Therefore, Hypothesis 2, which suggested that family demands would be positively related to FWC, received limited support.

**Table 4:** Multiple regression of the relationship between family demands and FWC

Step	Variable	*B*	*SE*	β	Δ*R* ^2^	*F* for Δ*R* ^2^
1	Controls					
	Gender	−0.01	0.19	0.00		
	Age	−0.02	0.01	−0.21*		
	Marital status	0.34	0.18	0.21	0.07	*F* (3, 130) = 3.10*
2	Family demands					
	Number of children	−0.06	0.05	−0.13		
	Number of older dependants	0.29	0.06	0.43**		
	Family time commitment	0.01	0.01	0.08	0.15	*F* (3, 127) = 8.14**
	Total *R* ^2^	0.22				
	*F* (6, 127)	5.88**				

Note

*p <0.05; **p <0.01.

Results of multiple regression analysis on the relationships of WFC and FWC with burnout are presented in Table 5. Together, WFC and FWC accounted for 20% of the explained variance in burnout beyond the control variables. As shown in this table, FWC (β = 0.40, *p* < 0.01) was positively related to burnout. However, WFC failed to significantly predict burnout (β = 0.13, *p* > 0.05). Therefore, the hypothesis that WFC and FWC would be positively related to burnout (Hypothesis 3) was partially supported.

**Table 5:** Multiple regression of the relationship between work–family conflict and burnout

Step	Variable	*B*	*SE*	β	Δ*R* ^2^	*F* for Δ*R* ^2^
1	Controls					
	Gender	−0.57	0.18	−0.24**		
	Age	−0.01	0.01	−0.16		
	Marital status	0.26	0.15	0.15	0.16	*F* (3, 130) = 8.36**
2	Work–family conflict					
	WFC	0.12	0.07	0.13		
	FWC	0.43	0.08	0.40**	0.20	*F* (2, 128) = 20.59**
	Total *R* ^2^	0.36				
	*F* (5, 128)	14.77**				

Note

WFC = work‐to‐family conflict; FWC = family‐to‐work conflict.

**p < 0.01.

## 5 DISCUSSION

The objectives of this study were to examine work and family demands as predictors of work–family conflict and the extent to which work–family conflict predicts job burnout among registered nurses in public health facilities in Ghana. We found long work hours to be associated with higher levels of WFC. The positive relationship between work hours and WFC is understandable since nurses spend long hours at work, with average work hours in this study being 44 hr per week. The low nurse‐to‐population ratio in Ghana often makes it necessary for nurses to work beyond the statutory 40 hr stipulated in Ghana's Labour Act (Act 651 of 2003). The present finding that long work hours are associated with high levels of WFC support previous studies (Adkins & Premeaux, 2012; Dugan et al., 2012) that reported positive relationships between work hours and work interference with family.

Our results also showed that increased frequency of weekend work was associated with higher levels of WFC. This finding is consistent with findings reported in previous studies (Fuß et al., 2008; Tammelin et al., 2017) that being called to work during weekend positively predicted work interference with family among physicians. Since nurses tend to spend high number of hours at work, it would be expected that weekends would provide opportunity to attend to family duties. Thus, working on weekend further detracted from time available for family responsibilities. Contrary to expectation, we did not find significant relationship between workload and WFC. This finding thus, contradicts previous studies (Fuß et al., 2008; Yildirim & Aycan, 2008) that reported positive relationship between workload and WFC among nurses. This finding also sharply contradicts a recent study among university employees in Ghana (Annor, 2016) that found a positive relationship between workload and WFC. Taken together, the present study suggests that long work hours and working on weekends have stronger influence of nurses' experience of WFC than level of quantitative workload.

The hypothesis that family‐related demands would predict FWC received limited support. Contrary to expectation, there was no significant relationship between number of children and FWC. This finding is at variance to previous studies that found positive relationship between number of children and interference of family with work (Byron, 2005; Grzywacz, 2000; Kinnunen & Mauno, 1998). An explanation for the present finding could be that number of children alone does not capture demands associated with caring for children. This is particularly noteworthy, given that in the Ghanaian context a high premium is placed on children and having large family size. This implies parents might not necessarily perceive high number of children as demanding. Moreover, older children may be a vital source of support to working parents in terms of their contribution to household labour (Annor, 2014). Similarly, time commitment to family‐related tasks was not significantly related to FWC. A possible explanation for this finding may be that time spent in the family domain is less structured, making it difficult for individuals to estimate the amount of time expended on family‐related tasks.

However, the number of older dependants at home was found to be a positive predictor of FWC. This finding contradicted a study (Boyar, Maertz, Pearson & Keough, 2003) that reported no significant relationship between caring for older parents and family interference with work. The present finding suggests that the cultural context may have an impact on the relationship between family demands and work–family conflict. In Ghana, due to the strong emphasis on the extended family, providing care for older people is regarded as a moral obligation on the part of their younger relatives (Oheneba‐sakyi & Takyi, 2006). Coupled with this expectation are limited coverage of social security benefits and absence of national policies that ensure provision of support for the aged (Annor, 2014). Consequently, older people become heavily reliant on their employed relatives for financial and material support, which increases pressure from the family domain among employees.

Findings from the present study also suggest differential impacts of WFC and FWC on burnout. Contrary to findings from previous research suggesting stronger relationship between WFC and burnout (Amstad et al., 2011; Reichl et al., 2014), we found that FWC positively predicted burnout while WFC was not significantly related to burnout. Thus, when nurses perceive demands from their family as interfering with their ability to perform work duties they are more likely to feel stressed as they struggle to meet job‐related demands, which may in turn result in burnout. According to Frone, Russell, and Cooper (1992), work–family conflict represents not just a source of stress but also a threat to constructing desirable role‐related image in other relevant life domains. In this regard, FWC “might represent a threat to constructing or maintaining a desired job‐related self‐image that has direct implications for an individual's overall sense of well‐being” (Frone et al., 1992, p. 74). This argument is relevant in the Ghanaian context, where work is viewed primarily as contributing to family welfare, a common construction of the meaning of work in most collectivistic societies (see Yang et al., 2000; Yang, 2005). The experience of FWC implies that employees might have difficulty performing effectively at work and since performance is linked to other desirable job outcomes such as promotion and its associated improvement in salary, nurses in Ghana may respond more negatively when family demands interfere with work duties.

This study has several limitations that need highlighting. First, the small size of the sample limits the extent to which findings from the study can be generalized. Second, the cross‐sectional nature of the study precludes making causal inferences based on the study's findings, while the reliance of self‐report single‐source data may raise concerns about common method bias. Studies using longitudinal designs based on relatively larger samples would be useful in establishing the temporal order of relationships among variables in the study and enhance generalizability of the results. Future research could mitigate concerns about common method bias by collecting data from multiple sources such spouses and co‐workers, which would also be useful for examining the possibility of crossover effects. Thirdly, the study focused on main effect relationships and did not examine possible moderators in the relationship between work/family demands and work–family conflict and between work–family conflict and burnout. For example, the level of importance nurses attach to their work and family roles could influence their perception of conflict between both roles and the level of support received at work and at home could influence whether nurses would experience burnout. Thus, additional research is needed to examine moderating variables in work–family conflict in this occupational setting. Finally, our reliance on contact persons in the selected health facilities to distribute the questionnaires may raise concerns that potential power imbalance between the contact persons and respondents could have influenced the response rate in the survey. We are, however, unable to estimate the extent of power imbalance inherent in the survey distribution approach and how that might have influenced the response rate.

In spite of these limitations, the present study suggests that work–family conflict has significant implications for individual nurses and health institutions due to its positive relationship with burnout. Considering the demonstrated negative effects of burnout on employee job attitudes and behaviours (Lee & Ashforth, 1996), it is important that organizations take steps to mitigate the effects of work–family conflict. Efforts by organizations to provide formal work–family support that helps employees achieve balance between work and family roles could demonstrate to employees that the organization cares about their family needs and thus, reduce work–family conflict and increase satisfaction with their jobs. This is particularly relevant to the Ghana Health Service as such policies could help improve retention of nurses in the service by making the profession more family‐friendly. The findings from the present study suggest that responsibilities associated with eldercare must be taken into consideration in formulating policies that would enable employees to achieve work–family balance.

In sum, this study has shown that work hours and weekend work are important predictors of WFC and having older dependants at home is associated with interference of family with work. The study also suggests that difficulty in integrating work and family roles is associated with higher levels of burnout. These findings demonstrate the need for policy to address workers' work and family needs particularly in the health sector. Overall, the study contributes to the nascent body of literature on the work–family interface in Ghana and sub‐Saharan Africa. It is hoped that the study will stimulate further research on individuals' experiences of combining work and family responsibilities in other occupational settings in Ghana.

## CONFLICT OF INTEREST

The authors declare no conflict of interest in publishing this study.

## AUTHOR CONTRIBUTIONS

The first author designed the study and carried out fieldwork. All authors were involved in the analysis and interpretation of results and writing of the manuscript. All authors read and approved the final manuscript.

All authors have agreed on the final version and meet at least one of the following criteria (recommended by the International Committee of Medical Journal Editors [https://www.icmje.org/recommendations/]):

- substantial contributions to conception and design, acquisition of data, or analysis and interpretation of data;
- drafting the article or revising it critically for important intellectual content.
